# Fatal Case of Strangulation of the Intestine

**Published:** 1844-04-20

**Authors:** Wm. M. M’Pheeters

**Affiliations:** St. Louis, Mo.


### FATAL CASE OF STRANGULATION OF THE INTESTINE.

By Wm. M. M'Pheeters, M.D., of St. Louis, Mo.

S. C., aged 44 years, of large frame, and, when in health, unusually athletic, had been under treatment for more than a year past, for what was supposed to be scirrhus of the intestines. Such remedies were administered as were best calculated to palliate the symptoms, and put off a fatal termination of the disease as long as possible.

*Feb.* 12.—He seemed unusually well, ate a hearty breakfast, and walked about during the forenoon. About noon he was taken suddenly with an excruciating pain in the lower part of the abdomen, which resisted all treatment, and continued to increase in severity until twelve o'clock at night, when he expired, having suffered from the time of his attack the most intense agony. He manifested throughout no disposition to vomit. Three or four days before his death he observed to his wife, while lying in bed, that he felt an unusual sensation in the abdomen, as though his intestines were *tied in a knot;* from this time he had no operation from his bowels, yet he complained of no pain until noon of the day of his death.

*Necroscopic Examination eleven hours after Death.*

Assisted by Drs. Pallen and Pope, a large circular flap, adherent above, was cut out of the abdomen, exhibiting its contents in sitû. On removing this flap our attention was arrested by the very dark red, almost black appearances of the ileum, resembling in color, the spleen when highly engorged with blood. The appearance extended not only throughout all the coats of the intestine, but also to the mesentery.

On examination it was discovered that this congestion was owing to a complete strangulation of the intestine, caused by the ileum making *three entire revolutions on itself*, with the peritoneum as an axis, thus preventing not only the passage of the fæcal matter, but also entirely arresting the circulation of the blood.

The lower part of the ileum, nine inches from the caput coli, was the first point of strangulation; seven inches above this the ileum passed around the latter point, or rather they were several times twisted around each other. The ileum then dipped down into the cavity of the pelvis, parallel with the rectum, and crossing it opposite the promontory of the sacrum. About fifteen inches above the last point of strangulation the ileum again passed through and around the other points,—thus causing a *double bow-knot*, very tightly drawn together, with two knuckles of intestine, one three and a half, and the other seven and a half inches long, greatly distended with gas.

The portion of the ileum which was strangulated seemed to be confined in the pelvis opposite the promontory of the sacrum, though we could discover no adhesions or membranous bonds. From twelve to fifteen feet of the intestine was involved in the congestion, extending upwards from the point first mentioned.

The several points where the intestine passed around itself were of a pale, almost white, color, presenting a marked contrast with the adjoining parts.

After the intestines were removed it was with some difficulty that the *twist* and *knot* could be relieved by pulling the knuckles through and untwisting.

The mesenteric glands of both the large and small intestines were considerably enlarged, and there was extensive scirrhous degeneration of the rectum, extending from the anus upwards six inches. Lungs not examined. The other viscera presented no marks of peculiar interest.

*Remarks.*—This case resembles somewhat that of the lamented Mr. Legare, late Attorney General of the United States, mentioned in the Boston Medical and Surgical Journal of last summer, although in his case the severity of the symptoms lasted for several days.

It is a curious matter of speculation how the intestines become tied up in the manner we sometimes find them. With the intestines attached to the mesentery and lying before him, I doubt whether the most ingenious manipulator could succeed in producing such a knot as was found in the present case; and yet nature effects it, we know not how.

*St. Louis, Mo.*, March 16th, 1844.

